# The Circadian Physiology: Implications in Livestock Health

**DOI:** 10.3390/ijms22042111

**Published:** 2021-02-20

**Authors:** Hao Li, Kaiqi Li, Kexin Zhang, Yanwei Li, Haotian Gu, Haoyu Liu, Zhangping Yang, Demin Cai

**Affiliations:** 1 Key Laboratory of Animal Breeding Reproduction and Molecular Design for Jiangsu Province, College of Animal Science and Technology, Yangzhou University, Yangzhou 225009, China; 17633532469@163.com (H.L.); kira_li@foxmail.com (K.L.); kitty20010606@126.com (K.Z.); lyw080805@163.com (Y.L.); guhaotian1998@163.com (H.G.); 2 College of Animal Science and Technology, Yangzhou University, Yangzhou 225009, China; yzp@yzu.edu.cn

**Keywords:** circadian physiology, clock gene, lipid metabolism, livestock, melatonin

## Abstract

Circadian rhythms exist in almost all types of cells in mammals. Thousands of genes exhibit approximately 24 h oscillations in their expression levels, making the circadian clock a crucial regulator of their normal functioning. In this regard, environmental factors to which internal physiological processes are synchronized (e.g., nutrition, feeding/eating patterns, timing and light exposure), become critical to optimize animal physiology, both by managing energy use and by realigning the incompatible processes. Once the circadian clock is disrupted, animals will face the increased risks of diseases, especially metabolic phenotypes. However, little is known about the molecular components of these clocks in domestic species and by which they respond to external stimuli. Here we review evidence for rhythmic control of livestock production and summarize the associated physiological functions, and the molecular mechanisms of the circadian regulation in pig, sheep and cattle. Identification of environmental and physiological inputs that affect circadian gene expressions will help development of novel targets and the corresponding approaches to optimize production efficiency in farm animals.

## 1. Introduction

Circadian rhythms are endogenous autonomous oscillators of physiological activities. They are controlled by the circadian clock directly or indirectly in a 24 h cycle [1]. It is a deeply implemented system during evolution in living organisms for adaptation to a cyclic natural environment [2]. For instance, the metabolic processes synchronizing to the biological clock allows animals to ramp up the necessary functions for the upcoming feeding and adapt to changes in the length of the day and night. In addition to ambient light and food availability, the circadian system is also important in mediating the timing of seasonal reproduction and other yearly physiological fluctuations that are regulated by day-length. This is extremely relevant in livestock science, where we purposely manipulate the photoperiod for production and management. However, the effects on animal health and ecology are often overlooked.

**Figure 1:**
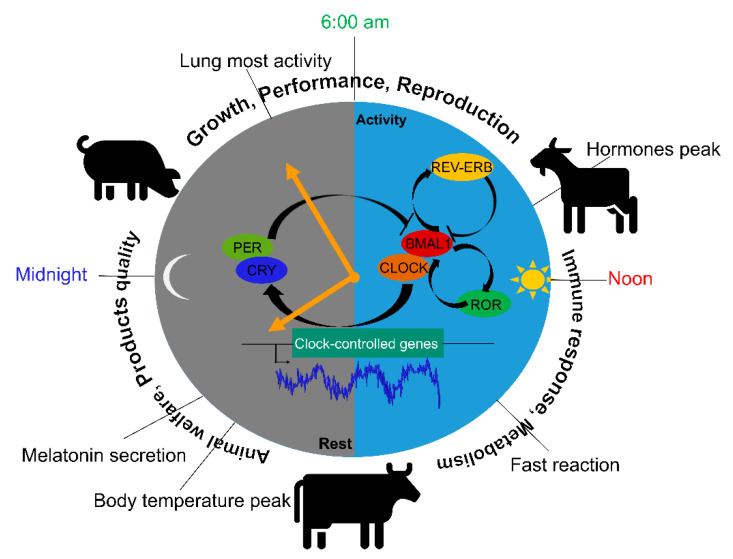
The circadian clock actions on physiology and health of livestock. Circadian rhythm plays a critical role in livestock’s growth performance, reproduction, metabolism, and the quality of their products. The core mechanism, the transcription–translation–feedback-loop (TTFL) drives the diurnal oscillations to maintain circadian activities. In which CLOCK and BMAL1 directly control the transcription of *PER* and *CRY* genes. Whilst PER and CRY proteins can repress their modulator, CLOCK and BMAL1-stimulated transcription occurs in a negative feedback loop, allowing the cycle to begin anew when PER and CRY actions are turned-over. Importantly, REV-ERBs and RORs dominate the *BMAL1* gene expression, are the primary players of the interlocked loop. In particular, hormones reach a peak level in the morning. Synthesis and release of melatonin is stimulated in the dark at night, while it is suppressed by light during the day. Loss of function of the circadian genes results in behavioral arrhythmicity, the disruption of the autoregulatory loop, and short period-length phenotypes.

The circadian clock exists in almost all types of cells in animals, and is based on an array of transcriptional regulators interacting with one another in negative feedback loops [2]. The rhythmicity of physiology is primarily controlled by the central clock, the suprachiasmatic nucleus (SCN) via sustaining the activity–resting and feeding–fasting cycles even under constant darkness [3]. This is by integrating the SCN oscillators of specific cell-type in different brain regions and the endocrine signals via synaptic and diffusible factors. In contrast, the peripheral tissues such as gut and liver are known to have their own autonomous circadian clocks. The peripheral clocks mediate metabolic responses and communicate constantly with the SCN [4,5]. Circadian rhythms are associated with many dietary factors that comprise different clock genes, and control various daily physiological events [6]. In mammals, the interlocked transcription–translation feedback loops act as a circadian pacemaker [7]. The basic helix–loop–helix transcription factors circadian locomotor output cycles kaput (CLOCK) and arnt-like protein-1 (BMAL1) make up the core component, directly control period (*PER*) and cryptochrome (*CRY*) transcription. Notably, PER and CRY proteins can inversely repress *CLOCK* and *BMAL1*-stimulated transcription, allowing the cycle to begin anew when *PER* and *CRY* actions are turned-over. Importantly, REV-ERBs (REV-ERBα and REV-ERBβ encoded by *NR1D1* and *NR1D2*, respectively) and retinoic acid receptor-related orphan receptors (RORs) drive the transcription of BMAL1, which are the primary players during the interlocked loop (Figure 1). Loss of function of these circadian genes results in behavioral arrhythmicity, the disruption of the autoregulatory loop, and short period-length phenotypes. In livestock production and intensive farming, circadian rhythms of domestic animals are vulnerable to factors such as transportation, breeding environment, antibiotics exposure, medication and feeding [8]. These lead to increased risks of many diseases, and subsequently huge economic losses in animal husbandry. Therefore, it is important to study in which way the circadian clock is associated with disease onset, and to unearth the underlying molecular mechanisms. The objective of this paper is to review the evidence of the rhythmic control of physiology and production in pigs and ruminants, and the associated clock gene regulations. A better understanding of the circadian physiology will help to show how rhythms could sustain livestock health and improve production.

## 2. The Circadian Physiology in Lipid Metabolism and the Clock Gene Regulate Action in Pigs

Circadian clocks are highly conserved endogenous oscillators. The central clock is entrained mainly by the light/dark cycle, whereas the peripheral circadian clocks exhibit tissue specificity and are entrained by a variety of environmental cues [3]. It is therefore context-dependent. Indeed, a wide range of physiological and metabolic variations depends on the circadian rhythm [9]. For instance, the lipid metabolism interconnecting with the circadian clock is essential for growth performance, health, and meat quality in pig production. Interestingly, Zhou and co-workers [10] have uncovered diurnal variations in polyunsaturated fatty acid (FA) metabolism in young pigs under physiological conditions. In circulation, the free FA is seen at its lowest level at 7 pm, and peaks at 3 am and 7 am the next day. While the plasma arachidonic acid, eicosapentaenoic acid, docosapentaenoic acid and docosahexaenoic acid (DHA) levels followed a similar trend. In contrast, the hepatic linoleic acid (LA) and α-linolenic acid are observed at higher concentrations in the daytime and lower at night, indicating an immediate absorption after meals during the day [10]. Surprisingly, the total triglycerides (TG) content in the liver of these young pigs did not show daily rhythm in the 24 h study, contrasting the previous observations in rodents [4]. As complex as this daily rhythm of lipid metabolism is reported to be in young animals, it can be reshaped by feeding patterns and nutrient supply. Compared to the conventional administration of three-meals, a dieting strategy of low to high protein contents as breakfast, lunch and dinner, respectively, results in the increased liver weight but decreased hepatic crude fat and reduced plasma TG levels in pigs. This is linked to a down-regulation of lipid metabolism genes, a reduced expression of *PER1*, *PER2* and *CRY2*, and an upregulation of BMAL1 protein expression in the liver [11]. More importantly, feeding according to the circadian system has been shown to improve the growth performance [12] and muscle quality of pigs [12,13], as well as yield better milk lipid profile and production in sows [14]. It appears that energy intake or feeding acts as a robust zeitgeber which synchronizes metabolism to animal peripheral clocks, especially for intensive pig farming. A group of circadian clock genes, i.e., *ARNTL*, *BHLHE*, *CRY2*, *NPAS2*, *NR1D1*, *PER1*, *PER2*, and *SIK1* [3] are shown to express in key metabolic tissues of pigs, including liver, small intestine, dorsal fat and muscle. Their expression levels differ significantly in pigs under fed states, compared to fasting [15]. In contrast, these genes are less affected in the central clock (hypothalamus). The feeding differences also influence the blood glucose and TG levels, which are shown to peak 2 h after eating and 4 h post-meal, respectively, and are accompanied by a significant decrease in free FA in blood [13]. We [16] have demonstrated previously that time-restricted feeding of limited nutrient supply reduces the cholesterol biosynthesis gene program in porcine liver organoids, a regulation that is controlled by RORγ [16,17,18].

As aforementioned, the timing of feed administration plays an important role in cooperating the metabolic responses to energy supply in animals. The nutritional outcomes can be very different, despite providing the same type of food/feed and the same amount of calories [19,20]. It is a true “time giver” to be manipulated in energy management of livestock and human beings, which is linked to the onset of obesity [21,22]. It is reported that a high-fat diet given during 12-h light causes more weight gain in mice than during 12-h darkness [23]. It should be noted that the mice are nocturnal, differing from pigs and humans that are diurnal [24]. More importantly, the light-phase feeding is indeed a common measure in the pig and poultry industries, and therefore is of high relevance. It requires more attention in regard to animal health, and studies of the obesity pathogenesis. Although many aspects of the circadian physiology are controlled by rhythmic input signals from the central clock, they also exhibit cell-autonomous regulation from the peripheral clocks [25]. A two-meals of low-high calcium supplementation dietary regimen is reported to influence the lipid metabolism of a pregnant sow during the third trimester [26]. In this study, the placenta lipid profile is also altered. i.e., the placental LA and DHA concentrations are increased by the high-low calcium feeding. In contrast, the heptadecanoic acid, oleic acid and monounsaturated fatty acid (MUFA) levels are decreased, compared to the low–high group, indicating a transfer of maternal effects [26]. This is mediated by changes of gene expression related to fetal lipid metabolism including FA de novo synthesis, transport, and glycolipid metabolism, as well as key clock genes *PER1*, *PER2* and *CLOCK* in placenta [26]. Collectively, it is suggested that feeding as a whole plays an essential role in circadian physiology of pigs. It is therefore important to regulate nutrition according to the peripheral circadian clocks.

For livestock, reproductive and growth performances are important meters. Wang et al. [27] reveal that the clock gene *BMAL1* plays a critical role in hormone secretion and apoptosis in porcine granulosa cells through the PI3K/AKT/mTOR pathway. This is closely related to oocyte maturation and follicular development [27]. Furthermore, it is reported that the carbon monoxide (CO) infusion in ophthalmic venous blood alters the clock gene expression (*PER1*, *PER2*, *CRY1*, *CRY2*, REV-ERBs/(*NR1D1/2*)) and their transcriptional factors (*BMAL1*, *NPAS2*, *CLOCK*, *RORβ*) in pigs. In parallel, CO treatment modifies the protein expression of the melatonin synthesis pathway [28]. Melatonin is known to be involved in synchronizing the circadian rhythm, and is especially linked to seasonal reproduction; it is therefore is a potent mediator of the timing and the associated physiological responses [29]. Indeed, melatonin addition has been demonstrated to prevent oocyte senescence after ovulation in pigs [30]. It upregulates the gene expression related to FA oxidation and mitochondrial biogenesis, and increases FA contents, ATP and mitochondria in porcine oocytes, which thereby provides an energy source for oocyte maturation and subsequent embryonic development [31]. This evidence of rhythmic clock gene expressions in the reproductive tissues suggests that they may contribute to fertility optimization [32]. In addition, biological rhythm acting on hormones such as catecholamines and glucocorticoids is shown to be associated with changes of the sympathetic stress. As observed in the sows, urinary catecholamine concentrations are lower in the dark, compared to during the daytime. Chronic activation of the sympathetic nervous system and the hypothalamic–pituitary–adrenal (HPA) axis is known to be associated solely with the increased catecholamines and glucocorticoids levels [33]. Their concentration in urine is thus a potential biomarker that helps to better understand hormone rhythmic release in circulation. As a non-invasive practice in pig husbandry [33], it could be applied to monitor the physiological changes of sows during pregnancy. The circadian physiology has been intensively studied in mice; less is known about the molecular components of these clocks and the mechanism by which they respond to external stimuli in domestic species [15]. Given the close physiological similarity between pigs and humans, data presented here could be also of interest for mechanistic studies in human physiology.

## 3. The Circadian Clock Gene Expression and the Associated Physiological Activities in Sheep

Seasonal reproduction is common among ruminants at all latitudes, including sheep and goats [34]. They breed during fall and are therefore called short-day (SD) breeders. In contrast, the long-day (LD) response is defined mainly by the decreased nightly duration, such as in summer [34]. By exposing the SD breeder Soay sheep to a long photoperiod of 16-hr light, waveforms of the SCN gene expressions were detected, similar to the observations in LD breeders. Accordingly, the expression of clock genes *PER1*, *PER2* and *FBXl21* were increased and were comparable to the levels in LD breeders [35]. Furthermore, Wagner and co-works [36] revealed a strong effect on estrus activity by acclimating the Soay sheep to LD photoperiods. In the SCN of sheep, a 24 h rhythm of *CLOCK* expression is detected along with *BMAL1* expression, exhibiting the features in anti-phase with cycles in *PER1* and *PER2* along with low-amplitude oscillation of *CRY1* and *CRY2* [37]. The waveform of only *PER1* and *PER2* expression is affected by photoperiod, with extended elevated expression under LD. In the hypothalamus, the expression of the kisspeptin-1 (*KISS-1*) gene that encodes kisspeptin, is known to be crucial for reproductive activation in sheep and is declined with transfer to long photoperiod [36]. Maximal *KISS-1* expression is observed in the 8:16 setting (8 h light, 16 h darkness). It has a strong suppressive effect on *KISS-1* expression, when shift to the 16:8 photoperiod (16 h light, 8 h darkness) [38]. The inducible cAMP early repressor (*ICER)* expression is significantly increased from zeitgeber time (ZT) 23 (6 am) to ZT3 (10 am), 3-h into the next light phase in the pars tuberalis (PT). In newborns, the expression levels of clock gene *PER1*, *PER2*, *CRY2* and *CLOCK* are higher at 8 am than at 8 pm, while the values of *BMAL1* are highest at 2 pm [39,40].

Plasma melatonin rhythms are seen to be synchronized to various light/dark cycles, while prolactin secretion is increased 4-5 times in response to the 16-h light/dark cycle, compared to SD photoperiod. The photoperiod shifts also affect the expression of circadian (*PER1*) and neuroendocrine output (*TSH*) genes in the PT, and the kisspeptin gene expression in the arcuate nucleus of the hypothalamus [36]. Another study in sheep points out that the rapid alternation of photoperiod impairs both the central (e.g., melatonin) and peripheral rhythmicity (e.g., skeletal muscle clock gene expressions), and disrupts the glucose homeostasis in sheep [41]. The blood glucose concentration is shown to decrease in response to the fast-alternate photoperiod environment, with the peak appearing in the daytime and the lowest point at daybreak. Together, this may reflect an adaptive mechanism of sheep related to the timing of the seasonal transitions via the hypothalamic–pituitary–gonadal pathway. As aforementioned, melatonin mediates the photoperiod alteration induced changes of clock gene expression, exhibiting an inversed oscillation of *PER1* and *PER2* expression. Moreover, the diurnal rhythm of melatonin secretion in sheep is different from *CRY1* expression, which is shown to increase from day to night [37]. In adult ewes, the mRNA levels of muscle *BMAL1* and *CLOCK* reach the peak at 6 pm during the early dark phase [41]. In contrast, the expression of *NR1D1* is found to peak at 0 h, whereas *PER3* and *CRY2* reach their highest expression levels at 6 am [41]. When the photoperiod alters rapidly, the peaking expressions of *BMAL1* and *NR1D1* are moved hours ahead during the day, compared to the control group. The expression of *PER1* at ZT0 (10 am) and ZT6 (4 pm) are decreased, and the expression of *NR1D2* at ZT12 (10 pm) is reduced [41]. In the ovine liver, the LD photoperiod induces a similar pattern of *PER2* and *BMAL1* expression (peaking at the end of the night), whereas under SD photoperiod *PER2* peaks in the early night opposite to BMAL1 and coincides with cortisol changes in blood [42]. Together, it is shown that these key genes regulate the normal operation of the circadian clock in sheep through their rhythmic expression.

In addition to ambient light, the seasonal transition also entails changes of environmental temperature, precipitation and other factors that all come down to the orbiting of the earth around the sun [34]. Indeed, diurnal variations are found in a wide range of physiological processes in sheep. For instance, the diurnal rhythm of liver glycogen concentration is significant, which is increased from dawn to dusk (at most 14 h after lighting) and decreased at night. The peak of plasma leptin during the day occurs in the middle of the light period (8 h after the lights come on), while the lowest level is reached after another 12 h (4 h before the lights went on) [43]. Remarkably, ewes are reported to exhibit strong rhythmicity in locomotor activity, body temperature, melatonin and glucose levels in circulation [44]. Their locomotor activity peaks at noon and drops to the lowest level at 8 pm, inversely related to melatonin secretion, plasma glucose level and body temperature. It is suggested that the melatonin rhythm in particular can be a valid phase marker of the circadian system in domestic species including sheep [29,44]. The relationship between melatonin secretion and the circadian clock was further addressed in lambs born to the melatonin-inhibited ewes. These newborns had less brown adipose tissues, and showed an exaggerated skin temperature response at 4 °C. It is shown that the expression of both thermogenic genes and the clock genes *BMAL1*, *CLOCK*, and *PER2* were increased due to their lacking melatonin, whereas a simple supplementation could reverse the course [45]. This is because the lambs have low arrhythmic endogenous levels of melatonin due to pineal gland immaturation during the first couple of weeks of life. Therefore, it is vital to maintain a normal daily rhythmic transfer of maternal melatonin to the fetus during gestation [46,47]. Disrupting this melatonin rhythm in neonatal lambs results in a decreased heart/body weight ratio and a suppression of plasma adrenocorticotropic hormone levels, as well as a downregulation of clock gene expression [39].

Interestingly, the proliferation of hair follicle stem cells is also affected by the circadian clock during the growth period, thus affecting the hair follicle cycle of goats [35]. In sheep, appetite and growth performance are closely related to the seasonal transition, by their changing the of expressions of the appetite regulatory peptide neuropeptide Y and agouti-related protein in the arcuate nucleus, as well as orexin in the lateral hypothalamus in response to photoperiod variations [48,49]. In summary, the hormone secretion, metabolism, growth, and reproduction of sheep are tightly regulated by the circadian clock. It is worth mentioning that, in addition to giving rise to meat, wool/hair, milk and skin products, sheep are also commonly used as a large diurnal animal model for human studies [41].

## 4. The Circadian Physiological Functions in Cattle

Environmental factors including photoperiod, heat and nutrition influence a series physiological processes of cattle. For instance, the estrus cycle of cows is related to the circadian clock [50]. In particular, diurnal oscillation is demonstrated in the secretion of luteinizing hormone (LH) in heifers. Of the 8-h cycle, i.e., 2 am to 9 am, 10 am to 5 pm, and 6 pm to 1 am, the LH preovulatory surge peaks 9, 3, and 1 time, respectively. The maximum average LH concentration is detected at 7 am. Thus, the peak before ovulation is more likely to appear between 2 am and 9 am [51]. In bull, the expression of clusterin genes *CLU* and *CLOCK* in semen was investigated. Both genes have been implicated in the functional attribution of male reproductive process. It is shown that *CLOCK* is expressed in higher level in good quality bull semen than in motility-impaired semen, whereas the expression of *CLU* displays an opposite manner [52]. It should be noted that no direct correlation or cause–effect relationship is revealed by this study, which therefore requires further investigation.

The circadian rhythm also modulates the production performance of cattle. First, it is shown that around 7% of the genes are expressed during lactation of cows that exhibit circadian patterns including core clock gene *BMAL1* and metabolic genes (e.g., *SREBP2*) [53]. The photoperiod of 16 h-light and 8 h-darkness is shown to enhance milk yield in a cost-effective manner, when compared to a short day of light exposure (8 h-light and 16 h-dark) [54,55]. During the dry period, the short day of light exposure seems to improve the subsequent lactation performance of dairy cows, possibly due to changes in mammary cell proliferation and metabolism [56]. In addition, silencing *PER2* gene in bovine mammary cells in vitro reveals that this clock genes regulate milk protein synthesis involving the mRNA expressions of casein-alpha s1 (*CSN1S1*) and casein-alpha s2 (*CSN1S2*) [57].

A long-day of light exposure also induces changes of the endocrine levels including increased prolactin and decreased melatonin in the blood of heifers and dairy cows [54,55]. In contrast, melatonin supplementation could reduce the serum prolactin level and mammary parenchymal growth induced by LD of light exposure in prepubertal heifers [58]. In addition to mimicking the photoperiodic effects on mammary growth, exogenous melatonin administration causes a significant increase in total cholesterol and TG content, as well as increasing the glucose and insulin levels, and reducing free FA concentrations in blood [59]. Indeed, as discussed in pig circadian physiology, melatonin is an important mediator, linking lipid metabolism and the circadian regulation. Finally, the feeding regime induces the profound effects on the diurnal variations in metabolism, as the peak blood glucose rhythm under the ad libitum condition is always earlier than the one-meal or two-meals daily feeding [60]. While nocturnal feeding to dairy cows leads to a reset of animal physiology, including the increased plasma insulin concentration, and decreased preformed FA, feed intake and digestibility of total dry matter and fiber [61,62].

Notably, domestic animal species including cattle exhibit the circadian variation of heart rate (HR) and arterial blood pressure [63]. It has been demonstrated that the cardiovascular functions of non-lactating pregnant dairy cows exhibit a diurnal periodicity in summer, and seasonal differences between summer and winter [64]. As the circadian clock is a measurement of the day/night length, the seasonal phenomenon can be considered as a response to changes of day length [63]. Nevertheless, it is shown that in these non-lactating cows, cardiac autonomic activities including HR variability in time and frequency display a significant diurnal rhythm in summer [64]. During this time, the cardiac autonomic function changes; for instance the HR increases from 7 am to 5 pm during the day, then declines from 6 pm to 6 am, showing a fluctuation among the four periods of the day [64]. A deeper understanding of the rhythmic pattern of the cardiovascular functions in ruminants may be useful for clinical, pharmacological purposes and physical performances in general.

Genes key to the circadian system that are linked to reproductive performance and their diurnal variations in cattle are discussed, as well as the effects on muscle functions and immunity etc. The transcripts of all circadian rhythm genes exist in the form of maternal mRNA, while the transcripts of *CLOCK*, *CRY1*, and *PER1* are abundant in oocytes, but are degraded immediately after fertilization [65,66,67,68]. The transcripts of *PER1*, *CRY1* and *CLOCK* are highly expressed in germinal vesicle and second metaphase oocytes of cattle, while *CRY2* and *PER2* transcripts are about ten times that of *BMAL1* [69]. Knockdown of the *CLOCK* gene inhibits the production of cow estradiol and the expressions of LH receptor and P450 aromatase mRNA in granulosa cells treated with follicle-stimulating hormone (FSH) [50]. Consistently, knockdown of *PER2* increases the production of progesterone and the expression of *STAR* mRNA in FSH treated granulosa cells and decreases the expression of LH receptor mRNA. The expression of *CLOCK* in healthy bulls is significantly higher than that of bulls with dyskinesias [52]. Exposure to dexamethasone treatment of bovine neutrophils decreased the mRNA expressions of *CLOCK* and *CRY1* and is associated with the increased *PER1* throughout the period. The decreased *NR1D1* and *CRY2* are observed at all times except 4:00, 16:00 and 24:00, respectively [70]. Recent research has only begun to reveal the complex but very interesting biological basis and ecological usefulness of the circadian clock and physiological rhythms in livestock, including those of the reproductive system which have both endogenous and exogenous influences and play important roles in cattle production [71].

## 5. Discussion

The photoperiod-affected diurnal rhythm of physiological activities in livestock comprising both central and peripheral clock regulations are different from those observations in rodents and are much less studied. Given that circadian oscillations play a pivotal role in the regulation of animal development, performance, reproduction and metabolism, how *i*. to exploit the biological clock to ensure the healthy growth of pigs, sheep and cattle; *ii*. to improve the livestock production efficiency remain an open issue worth further discussion.

### 5.1. Photoperiod-Affected Hormone Regulation

Notably, evaluation of the adaptive responses of pigs in practice is quite challenging. The main reasons include the collection of samples such as blood and how to examine functional neuroendocrine systems properly. For hormone measurement in pigs, corticosteroids levels in urine could become a noninvasive way to detect and reflect activity changes in the HPA axis and the sympathetic nervous system [33]. However, the urinary sampling from pigs is also accompanied with stress responses, especially for sensitive tests like adrenocortical function assay. It is largely dependent on specific management procedures. Cortisol levels, especially glucocorticoids exhibit a 24 h oscillation. This pattern of hormone matches the phasing of the hepatic circadian gene *PER2* expression, which together determines the hormone function observed in pigs and in sheep exposed to an LD photoperiod [42]. In dairy cow, it is well-accepted that the biological clock system is associated with metabolism and hormonal alterations; both are required for initiating and sustaining lactation [72]. Thus, maintaining normal circadian rhythm during these transition phases is critical for milk production and metabolic regulation in early lactation. Moreover, it has been addressed that the pineal gland plays an important role in controlling of gonadal atrophy in winter, by increasing melatonin secretion via the HPA axis in animals [73]. In birds, the pineal gland is also involved in the regulation of locomotor activity [74]. However, the regulatory functions of the pineal in pigs and the mechanisms behind it are not explored.

### 5.2. The Melatonin Rhythm

In livestock, melatonin secretion is a key hormonal indicator of day-length changes and seasonal difference. It represents an internal signal of the brain to reflect the external photoperiod [29,36]. As a seasonally breeding mammal, sheep is more sensitive to the circadian hormone signals, especially melatonin [63]. It produces long-duration signals in winter and transient signals in summer, therefore determining the seasonal differences of the circadian physiology [75]. Interestingly, it appears that the natural melatonin rhythm in various animal species has a time window to occur, as neonates lack the rhythmic secretion of melatonin. In lambs, high-amplitude melatonin exposure in early-life leads to the obvious alterations of the fetal-to-neonatal transition, which underlies the potential risk of melatonin administration during the neonatal phase [39]. We suggest that learning the functions and diurnal variations of the above-mentioned hormones in domestic species is necessary. More importantly, a better understanding of the molecular mechanisms of the circadian gene expression and how these complex hormonal signals are integrated into the circadian system will help to expand our knowledge and to generate novel targets and strategies in livestock management.

### 5.3. The Circadian Regulation of Seasonal Activity

Circadian clocks regulations are closely related to metabolic adaptations in multiple tissues. They control the core circadian gene transcriptional profiles in the mammary gland [57], liver [4,11] and adipose tissue [45] enrolled in the periportal period. The circadian physiology dominates the smooth transition of gestation to lactation in dairy cows. We propose that future research may focus more on how environmental cues like nutrition and feeding could entrain the diurnal oscillations in key metabolic organs via specific mechanisms. In line with the nutritional challenge in ruminants during the transition phase, lipid deposition is a critical event for pig production, especially during the fattening period. However very few studies have paid attention to the circadian modification of genes involved in lipid absorption, transport, storage, and catabolism [13]. With regard to meat quality, muscle metabolism and its relation to nutrient availability/feeding should be addressed to provide a better understanding of the genetic basis of energy homeostasis in pigs. Further experiments are therefore needed to determine the mechanistic actions of a specific nutrient coupling a specific clock gene expression and/or a particular physiological activity in pigs.

Maintaining the homeostasis of body temperature is crucial for cattle production. Environmental factors including the high-level ambient temperature, indoor or outdoor environment, thermal oscillations and solar radiation exposures all pose great challenges to animal husbandry in tropical regions [76]. The resultant changes could be long-lasting, showing a significant difference compared to that of temperate zones [77,78], suggesting meteorological variables in response to seasonal fluctuations. As discussed earlier, the cardiac autonomic activity of cattle exhibits a significant daily oscillation in summer compared to in winter. This could be attributed to high-level ambient temperature influencing endogenous signaling [64]. Further studies are needed to clarify interactions between other environmental factors including humidity, feeding patterns and the cardiac regulation and capacity in large animals, with an emphasis on the effects of circadian rhythm.

## 6. Conclusions

The interconnection between circadian rhythmicity and livestock physiology is becoming one of the major focuses of the field of animal science [5]. Indeed, the temporal organization of a 24 h cycle physiology, metabolism, and behavior is driven by a complex network of multiple cellular circadian oscillations. Clock genes and actions modulating circadian oscillation in livestock are summarized in Table 1. As expected, the pacemaker BMAL1 plays the dominant roles in all three livestock species we have discussed, i.e., pigs, sheep and cattle, and the action of BMAL1 is tightly connected with PER2 and CLOCK. The rhythmical pattern is synchronized through neuronal and hormonal signals by a core center-clock located in the suprachiasmatic nuclei zone of the hypothalamus. Studies on the regulations of circadian clock gene expression have so far been conducted almost exclusively using commercially available cell lines along with small experimental animals. In contrast, studies using livestock species (either domestic or wild-cross-breeding ones) for the assessment of circadian clock modulation are still rare and with limitations. Specific feeding of circadian time or chrono-treatment is important to animal science, and additional advances should be made in large animal chronobiology. We expect that future research into the cellular and molecular mechanisms that mediates circadian and seasonal influences on animal physiology (e.g., endocrinology and metabolism) and pathology will fundamentally improve the feeding application and management in animal husbandry. As the saying goes, it is about time.

## Figures and Tables

**Table 1:** Core genes of circadian rhythm and their functions in livestock.

Genes	Pigs	Sheep	Cattle
ARNTL (BMAL)	Crude protein intake of three meals regulates the protein expression [11].	Decreasing brown adipose tissue in neonates [45].	
	Promoting the secretion of progesterone and estradiol and inhibiting the apoptosis of granulosa cells [27].	Sensitized to long-term light or without melatonin [45].	Increasing the transcript levels of NR1D1 and Prostaglandin G/H synthetase 2 (PTGS2) in uterine stromal (USCs) [71]; Increasing the production of prostaglandin F_2_α (PGF_2_α) [71].
	Controlling the expression of ovarian genes in porcine granulosa cells [27].	Different expression peaks in long-day or short-day conditions [42].	
	Suppression of mRNA levels of circadian genes [27].	Rapidly alternating photoperiods causes 2.5-h earlier expression peak [41].	
CRY1	Reducing the expression of pro-inflammatory cytokines Interleukin-6 (IL-6) and Tumor-necrosis-factor alpha (TNF-α) [22].	High expression in night or along with high melatonin concentration [36,37,40].	
	Stabilizing the circadian oscillation and keeping the body temperature constant [22].		
PER2	Crude protein intake of three meals regulates the protein expression [11].	Decreasing brown adipose tissue in neonates [45];	Increasing the proliferation of granulosa cells treated with FSH [50];
	Calcium supplementation at different times inhibits mRNA expression in placenta [26].	Synchronizing with the plasma insulin and glucose concentrations [41,42].	Decreasing the progesterone production and expression of STAR mRNA in FSH-treated granulosa cells [50];
		long-term light or decreased melatonin reduces mRNA expression [37,45].	Enhancing αs–casein protein synthesis of bovine mammary epithelial cells (BMEC) and an overall increase in milk protein content [57].
		Different expression peaks in long-day or short-day conditions [42].	
PER1	Crude protein intake of three meals regulates the protein expression [11].	Increasing the prolactin secretion [36].	
	Calcium supplementation indicator [26].	High expression in night [37,40].	
	A transmitter of light signal. The expression in the PA and DH was elevated in the animals treated with CO [28].	In SCN, PER1 peaks early in the day [38].	
NR1D1		Decreasing the plasma insulin and glucose concentrations [41].	Repressing PTGS2 gene expression [71]
		In PT, melatonin inhibits mRNA expression [40].	
		Rapidly alternating photoperiods causes 2.5 h earlier expression peak [41].	
CLOCK	Calcium supplementation indicator [26].	Decreasing the plasma insulin and glucose concentrations [41].	Increasing estradiol production, and luteinizing hormone receptor (LHr) and P450 aromatase (P450arom) mRNAs in FSH-treated granulosa cells [50];

## Data Availability

Data sharing not applicable.

## References

[1] Reppert S.M., Weaver D.R. (2002). Coordination of circadian timing in mammals. Nature.

[2] Takahashi J.S., Hong H.-K., Ko C.H., McDearmon E.L. (2008). The genetics of mammalian circadian order and disorder: Implications for physiology and disease. Nat. Rev. Genet..

[3] Panda S. (2016). Circadian physiology of metabolism. Science.

[4] Adamovich Y., Rousso-Noori L., Zwighaft Z., Neufeld-Cohen A., Golik M., Kraut-Cohen J., Wang M., Han X., Asher G. (2014). Circadian Clocks and Feeding Time Regulate the Oscillations and Levels of Hepatic Triglycerides. Cell Metab..

[5] Cribbet M.R., Logan R.W., Edwards M.D., Hanlon E., Peek C.B., Stubblefield J.J., Vasudevan S., Ritchey F., Frank E. (2016). Circadian rhythms and metabolism: From the brain to the gut and back again. Ann. New York Acad. Sci..

[6] Patke A., Young M.W., Axelrod S. (2020). Molecular mechanisms and physiological importance of circadian rhythms. Nat. Rev. Mol. Cell Biol..

[7] Miller B.H., McDearmon E.L., Panda S., Hayes K.R., Zhang J., Andrews J.L., Antoch M.P., Walker J.R., Esser K.A., HogenEsch J.B. (2007). Circadian and CLOCK-controlled regulation of the mouse transcriptome and cell proliferation. Proc. Natl. Acad. Sci..

[8] Bae S.-A., Fang M.Z., Rustgi V., Zarbl H., Androulakis I.P. (2019). At the Interface of Lifestyle, Behavior, and Circadian Rhythms: Metabolic Implications. Front. Nutr..

[9] Asher G., Sassone-Corsi P. (2015). Time for Food: The Intimate Interplay between Nutrition, Metabolism, and the Circadian Clock. Cell.

[10] Zhou X., Wan D., Zhang Y., Zhang Y., Long C., Chen S., He L., Tan B., Wu X., Yin Y. (2017). Diurnal variations in polyunsaturated fatty acid contents and expression of genes involved in their de novo synthesis in pigs. Biochem. Biophys. Res. Commun..

[11] Xie C., Duan X., Long C., Wu X. (2020). Hepatic lipid metabolism is affected by a daily 3-meal pattern with varying dietary crude protein with a pig model. Anim. Nutr..

[12] Wu X., Xie C., Long C., Li J., Zhou X., Fan Z., Blachier F., Yin Y. (2017). Effects of a daily three-meal pattern with different dietary protein contents on pig growth performance, carcass and muscle quality traits. J. Sci. Food Agric..

[13] Cardoso T.F., Quintanilla R., Tibau J., Gil M., Mármol-Sánchez E., González-Rodríguez O., González-Prendes R., Amills M. (2017). Nutrient supply affects the mRNA expression profile of the porcine skeletal muscle. BMC Genom..

[14] Wu X., Xie C., Guo X., Long C., Zhang T., Gao T., Yin Y. (2017). A Maternal Two-meal Feeding Sequence with Varying Crude Protein Affects Milk Lipid Profile in A Sow-Piglet Model. Sci. Rep..

[15] Cardoso T.F., Quintanilla R., Castelló A., Mármol-Sánchez E., Ballester M., Jordana J., Amills M. (2018). Analysing the Expression of Eight Clock Genes in Five Tissues From Fasting and Fed Sows. Front. Genet..

[16] Zhang K., Li H., Xin Z., Li Y., Wang X., Hu Y., Liu H., Cai D. (2020). Time-restricted feeding downregulates cholesterol biosynthesis program via RORγ-mediated chromatin modification in porcine liver organoids. J. Anim. Sci. Biotechnol..

[17] Takeda Y., Kang H.S., Freudenberg J., DeGraff L.M., Jothi R., Jetten A.M. (2014). Retinoic Acid-Related Orphan Receptor γ (RORγ): A Novel Participant in the Diurnal Regulation of Hepatic Gluconeogenesis and Insulin Sensitivity. PLoS Genet..

[18] Cai D., Wang J., Gao B., Li J., Wu F., Zou J.X., Xu J., Jiang Y., Zou H., Huang Z. (2019). RORγ is a targetable master regulator of cholesterol biosynthesis in a cancer subtype. Nat. Commun..

[19] Manoogian E.N.C., Chaix A., Panda S. (2019). When to Eat: The Importance of Eating Patterns in Health and Disease. J. Biol. Rhythm..

[20] Deng Y., Wang Z.V., Gordillo R., An Y., Zhang C., Liang Q., Yoshino J., Cautivo K.M., De Brabander J., Elmquist J.K. (2017). An adipo-biliary-uridine axis that regulates energy homeostasis. Science.

[21] Garaulet M., Gómez-Abellán P. (2014). Timing of food intake and obesity: A novel association. Physiol. Behav..

[22] Liu H., Li Y., Wei Q., Liu C., Bolund L., Vajta G., Dou H., Yang W., Xu Y., Luan J. (2013). Development of Transgenic Minipigs with Expression of Antimorphic Human Cryptochrome 1. PLoS ONE.

[23] Arble D.M., Bass J., Laposky A.D., Vitaterna M.H., Turek F.W. (2009). Circadian Timing of Food Intake Contributes to Weight Gain. Obesity.

[24] Meurens F., Summerfield A., Nauwynck H., Saif L., Gerdts V. (2012). The pig: A model for human infectious diseases. Trends Microbiol..

[25] Liu A.C., Welsh D.K., Ko C.H., Tran H.G., Zhang E.E., Priest A.A., Buhr E.D., Singer O., Meeker K., Verma I.M. (2007). Intercellular coupling confers robustness against mutations in the SCN circadian clock network. Cell.

[26] Gao L.-M., Xie C.-Y., Zhang T.-Y., Wu X., Yin Y.-L. (2018). Maternal supplementation with calcium varying with feeding time daily during late pregnancy affects lipid metabolism and transport of placenta in pigs. Biochem. Biophys. Res. Commun..

[27] Wang W., Yin L., Bai L., Ma G., Zhao C., Xiang A., Pang W., Yang G., Chu G. (2017). Bmal1 interference impairs hormone synthesis and promotes apoptosis in porcine granulosa cells. Theriogenology.

[28] Gilun P., Stefanczyk-Krzymowska S., Romerowicz-Misielak M., Tabecka-Lonczynska A., Przekop F., Koziorowski M. (2013). Carbon monoxide-mediated humoral pathway for the transmission of light signal to the hypothalamus. J. Physiol. Pharmacol. Off. J. Pol. Physiol. Soc..

[29] Lincoln G.A., Andersson H., Loudon A. (2003). Clock genes in calendar cells as the basis of annual timekeeping in mammals--a unifying hypothesis. J. Endocrinol..

[30] Wang T., Gao Y.-Y., Chen L., Nie Z.-W., Cheng W., Liu X., Schatten H., Zhang X., Miao Y.-L. (2017). Melatonin prevents postovulatory oocyte aging and promotes subsequent embryonic development in the pig. Aging.

[31] Jin J.-X., Lee S., Taweechaipaisankul A., Kim G.A., Lee B.C. (2017). Melatonin regulates lipid metabolism in porcine oocytes. J. Pineal Res..

[32] Kennaway D.J. (2005). The role of circadian rhythmicity in reproduction. Hum. Reprod. Updat..

[33] Hay M., Meunier-Salaün M.-C., Brulaud F., Monnier M., Mormède P. (2000). Assessment of hypothalamic-pituitary-adrenal axis and sympathetic nervous system activity in pregnant sows through the measurement of glucocorticoids and catecholamines in urine. J. Anim. Sci..

[34] Shinomiya A., Shimmura T., Nishiwaki-Ohkawa T., Yoshimura T. (2014). Regulation of Seasonal Reproduction by Hypothalamic Activation of Thyroid Hormone. Front. Endocrinol..

[35] Dardente H., Wyse C.A., Lincoln G.A., Wagner G.C., Hazlerigg D.G. (2016). Effects of Photoperiod Extension on Clock Gene and Neuropeptide RNA Expression in the SCN of the Soay Sheep. PLoS ONE.

[36] Wagner G.C., Johnston J.D., Clarke I.J., Lincoln G.A., Hazlerigg D.G. (2008). Redefining the Limits of Day Length Responsiveness in a Seasonal Mammal. Endocrinology.

[37] Lincoln G., Messager S., Andersson H., Hazlerigg D. (2002). Temporal expression of seven clock genes in the suprachiasmatic nucleus and the pars tuberalis of the sheep: Evidence for an internal coincidence timer. Proc. Natl. Acad. Sci. USA.

[38] Johnston J., Bashforth R., Diack A., Andersson H., Lincoln G., Hazlerigg D. (2004). Rhythmic melatonin secretion does not correlate with the expression of arylalkylamine N-ACETYLTRANSFERASE, inducible cyclic amp early repressor, period1 or cryptochrome1 mRNA in the sheep pineal. Neuroscience.

[39] Seron-Ferre M., Torres-Farfan C., Valenzuela F.J., Castillo-Galan S., Rojas A., Mendez N., Reynolds H., Valenzuela G.J., Llanos A.J. (2017). Deciphering the Function of the Blunt Circadian Rhythm of Melatonin in the Newborn Lamb: Impact on Adrenal and Heart. Endocrinology.

[40] Johnston J.D., Tournier B.B., Andersson H., Masson-Pévet M., Lincoln G.A., Hazlerigg D.G. (2006). Multiple Effects of Melatonin on Rhythmic Clock Gene Expression in the Mammalian Pars Tuberalis. Endocrinology.

[41] Varcoe T.J., Gatford K.L., Voultsios A., Salkeld M.D., Boden M.J., Rattanatray L., Kennaway D.J. (2014). Rapidly alternating photoperiods disrupt central and peripheral rhythmicity and decrease plasma glucose, but do not affect glucose tolerance or insulin secretion in sheep. Exp. Physiol..

[42] Andersson H., Johnston J.D., Messager S., Hazlerigg D., Lincoln G. (2005). Photoperiod regulates clock gene rhythms in the ovine liver. Gen. Comp. Endocrinol..

[43] Chakir I., Dumont S., Pévet P., Ouarour A., Challet E., Vuillez P. (2015). The circadian gene Clock oscillates in the suprachiasmatic nuclei of the diurnal rodent Barbary striped grass mouse, Lemniscomys barbarus: A general feature of diurnality?. Brain Res..

[44] Piccione G., Caola G., Refinetti R. (2005). Temporal relationships of 21 physiological variables in horse and sheep. Comp. Biochem. Physiol. Part A: Mol. Integr. Physiol..

[45] Seron-Ferre M., Reynolds H., Mendez N.A., Mondaca M., Valenzuela F., Ebensperger R., Valenzuela G.J., Herrera E.A., Llanos A.J., Torres-Farfan C. (2015). Impact of Maternal Melatonin Suppression on Amount and Functionality of Brown Adipose Tissue (BAT) in the Newborn Sheep. Front. Endocrinol..

[46] Nowak R., Young I.R., McMillen I.C. (1990). Emergence of the diurnal rhythm in plasma melatonin concentrations in newborn lambs delivered to intact or pinealectomized ewes. J. Endocrinol..

[47] Kennaway D.J., Stamp G.E., Goble F.C. (1992). Development of melatonin production in infants and the impact of prematurity. J. Clin. Endocrinol. Metab..

[48] Archer Z.A., Findlay P.A., Rhind S.M., Mercer J.G., Adam C.L. (2002). Orexin gene expression and regulation by photoperiod in the sheep hypothalamus. Regul. Pept..

[49] Clarke I.J., Rao A., Chilliard Y., Delavaud C., Lincoln G.A. (2003). Photoperiod effects on gene expression for hypothalamic appetite-regulating peptides and food intake in the ram. Am. J. Physiol. Integr. Comp. Physiol..

[50] Shimizu T., Hirai Y., Murayama C., Miyamoto A., Miyazaki H., Miyazaki K. (2011). Circadian Clock genes Per2 and clock regulate steroid production, cell proliferation, and luteinizing hormone receptor transcription in ovarian granulosa cells. Biochem. Biophys. Res. Commun..

[51] Ginther O., Pinaffi F., Khan F., Duarte L., Beg M. (2013). Circadian influence on the preovulatory LH surge, ovulation, and prolactin concentrations in heifers. Theriogenology.

[52] Kumar S., Deb R., Singh U., Ganguly I., Mandal D.K., Tyagi S., Kumar M., Sengar G., Sharma S., Singh R. (2015). Bovine Circadian Locomotor Output Cycles Kaput (CLOCK) and Clusterin (CLU) mRNA Quantitation in Ejaculated Crossbred Bull Spermatozoa. Reprod. Domest. Anim..

[53] Plaut K., Casey T. (2012). Does the circadian system regulate lactation?. Animal.

[54] Peters R., Chapin L., Leining K., Tucker H. (1978). Supplemental lighting stimulates growth and lactation in cattle. Science.

[55] Peters R., Chapin L., Emery R., Tucker H. (1981). Milk Yield, Feed Intake, Prolactin, Growth Hormone, and Glucocorticoid Response of Cows to Supplemented Light. J. Dairy Sci..

[56] Dahl G.E. (2008). Effects of short day photoperiod on prolactin signaling in dry cows: A common mechanism among tissues and environments?. J. Anim. Sci..

[57] Hu L.Y., Wang M.Z., Ouyang J.L., Li P.F., Loor J.J. (2017). Rapid Communication: Period2 gene silencing increases the synthesis of alphas-casein protein in bovine mammary epithelial cells. J. Anim Sci..

[58] Sanchez-Barcelo E.J., Mediavilla M.D., Zinn S.A., A Buchanan B., Chapin L.T., Tucker H.A. (1991). Melatonin Suppression of Mammary Growth in Heifers1. Biol. Reprod..

[59] Darul K., Kruczyńska H. (2004). Effect of melatonin on biochemical variables of the blood in dairy cows. Acta Veter- Hung..

[60] Piccione G., Fazio F., Caola G., Refinetti R. (2008). Daily Rhythmicity of Glycemia in Four Species of Domestic Animals under Various Feeding Regimes. J. Physiol. Sci..

[61] Niu M., Harvatine K. (2018). Short communication: The effects of morning compared with evening feed delivery in lactating dairy cows during the summer. J. Dairy Sci..

[62] Niu M., Ying Y., Bartell P., Harvatine K. (2014). The effects of feeding time on milk production, total-tract digestibility, and daily rhythms of feeding behavior and plasma metabolites and hormones in dairy cows. J. Dairy Sci..

[63] Piccione G., Grasso F., Giudice E. (2005). Circadian rhythm in the cardiovascular system of domestic animals. Res. Veter- Sci..

[64] Kovács L., Kézér F.L., Ruff F., Szenci O. (2016). Cardiac autonomic activity has a circadian rhythm in summer but not in winter in non-lactating pregnant dairy cows. Physiol. Behav..

[65] Amano T., Matsushita A., Hatanaka Y., Watanabe T., Oishi K., Ishida N., Anzai M., Mitani T., Kato H., Kishigami S. (2009). Expression and Functional Analyses of Circadian Genes in Mouse Oocytes and Preimplantation Embryos: Cry1 Is Involved in the Meiotic Process Independently of Circadian Clock Regulation1. Biol. Reprod..

[66] Oh B., Hwang S.Y., Solter D., Knowles B.B. (1997). Spindlin, a major maternal transcript expressed in the mouse during the transition from oocyte to embryo. Dev..

[67] Gosden R.G. (2002). Oogenesis as a foundation for embryogenesis. Mol. Cell. Endocrinol..

[68] Hanrahan J.P., Gregan S.M., Mulsant P., Mullen M., Davis G.H., Powell R., Galloway S.M. (2004). Mutations in the Genes for Oocyte-Derived Growth Factors GDF9 and BMP15 Are Associated with Both Increased Ovulation Rate and Sterility in Cambridge and Belclare Sheep (Ovis aries)1. Biol. Reprod..

[69] Amano T., Tokunaga K., Kakegawa R., Yanagisawa A., Takemoto A., Tatemizo A., Watanabe T., Hatanaka Y., Matsushita A., Kishi M. (2010). Expression analysis of circadian genes in oocytes and preimplantation embryos of cattle and rabbits. Anim. Reprod. Sci..

[70] Nebzydoski S.J., Pozzo S., Nemec L., Rankin M.K., Gressley T.F. (2010). The effect of dexamethasone on clock gene mRNA levels in bovine neutrophils and lymphocytes. Veter- Immunol. Immunopathol..

[71] Isayama K., Chen H., Yamauchi N., Hattori M.A. (2014). REV-ERBalpha inhibits the PTGS2 expression in bovine uterus endometrium stromal and epithelial cells exposed to ovarian steroids. J. Reprod. Dev..

[72] Casey T.M., Plaut K. (2012). LACTATION BIOLOGY SYMPOSIUM: Circadian clocks as mediators of the homeorhetic response to lactation1. J. Anim. Sci..

[73] Reiter R.J. (1973). Comparative Physiology: Pineal Gland. Annu. Rev. Physiol..

[74] Turek F.W., McMillan J.P., Menaker M. (1976). Melatonin: Effects on the circadian locomotor rhythm of sparrows. Science.

[75] Dupré S.M., Loudon A.S. (2007). Circannual Clocks: Annual Timers Unraveled in Sheep. Curr. Biol..

[76] Nascimento S.T., Maia A.S.C., Fonsêca V.D.F.C., Nascimento C.C.N., De Carvalho M.D., Pinheiro M.D.G. (2019). Physiological responses and thermal equilibrium of Jersey dairy cows in tropical environment. Int. J. Biometeorol..

[77] Da Silva R.G., Guilhermino M.M., de Morais D.A. (2010). Thermal radiation absorbed by dairy cows in pasture. Int. J. Biometeorol..

[78] Da Silva R.G., Maia A.S., de Macedo Costa L.L., de Queiroz J.P. (2012). Latent heat loss of dairy cows in an equatorial semi-arid environment. Int. J. Biometeorol..

